# Some General Principles in the Treatment of Fracture Cases

**Published:** 1908-11-07

**Authors:** 


### Resident Medical Officers' Department.

### SOME GENERAL PRINCIPLES IN THE TREATMENT OF FRACTURE CASES.

In few of his duties is the house surgeon, speaking generally, allowed by his visiting surgeon such unfettered discretion as in the management of fracture cases. For some reason or other, possibly because they are among the few patients in surgical wards on whom operation is not necessary, a great many (though, of course, not all) visiting surgeons take less interest in the subjects of fracture than in the other inmates of their wards. In consequence of this the resident medical officer then has unique opportunities of perfecting himself in the details of setting, splinting, and after-treatment generally of these cases. There is always the danger that he may seize the opportunity to avoid hard labour in his accident ward by deputing his proper responsibilities to the sister and nurses, but so short-sighted a policy is absolutely certain to cause him acute regret subsequently.

To the really keen man the knowledge that to him is being left the treatment of cases so immensely important is a stimulus to the most unremitting care and attention, by which alone can satisfactory results be obtained. At first sight it looks so simple to apply the regulation splints advised in text-books for particular fractures, and so easy to walk down the ward each morning to ask the wearers of these splints if they are comfortable; but in reality there is no class of surgical case in which constant supervision is so much required and so well repaid. It is said that a man may be known by the company he keeps: certainly a house surgeon is best estimated by the results of his fracture cases and by the verdict of the sister of his accident ward.

### When to Set Fractures.

There are many points in connection with the (non-operative) treatment of simple fractures about which unanimity cannot be said to prevail either among house surgeons or their senior colleagues. The opinions of an individual are bound to be coloured to some extent by the practice of the medical school in which he has been educated, for custom and tradition exert a very powerful influence in these matters. One often-discussed question is the proper time at which to "put up" an ordinary simple fracture, say, of both bones of the leg. Some have advised immediate reduction and the appropriate splinting; others still maintain that the better way is to make the patient comfortable for twelve, twenty-four, or even thirty-six hours by means of a rolled junk before attempting to confine the limb within any restrictive apparatus.

The advocates of the first method argue that the sooner the parts are restored to their normal position the less is the damage to the tissues, and the sooner the reparatory processes commence: those who follow the second method base their preference on the argument that extravasation and œdema should be allowed their full development before any tight splints or bandages are applied. The trend of present-day practice seems to be rather in favour of the immediate treatment, but it must be admitted that there is something to be said for the other plan.

Closely connected with the foregoing is the necessity or otherwise of employing general anæsthesia in hospital for the purpose of setting a fracture. If it is decided to do so it is often advisable to postpone it for a few hours, especially in the not uncommon case of a powerful man who has broken a limb after a luxus consumption of alcohol, together with a hearty meal. The convenience to the surgeon of an unconscious and immobile patient with relaxed muscles is so great that it is becoming nearly a universal rule to set fractures under anæsthesia. While convinced that this procedure is for most cases absolutely in the best interest of the patient also, the writer would lay it down that cases should be treated on their merits, and that there are a fair proportion of these patients for whom anæsthesia is quite unnecessary, and, therefore, contra-indicated. There is a certain danger attending the routine use of anæsthetics, and this is that the house surgeon may acquire the habit of using too much force and too little skill in coapting the ends of a fracture: even in children it is often possible by gentle handling to obtain satisfactory alignment without resorting to anæsthesia.

### Fractures and Alcohol.

To the question of alcoholic excess allusion has already been made. It has been calculated at a large general hospital that of the admissions for accidents, about 70 per cent. are in those who have "been drinking," and more than a third of these are persons helplessly drunk. It is, therefore, no matter for wonder that alcoholic delirium is so common in accident wards, setting in generally about three days after the patient's admission. Whether the opinion is accurate, that the onset of this complication is partly provoked by the sudden abstinence from alcohol which naturally follows entrance into a hospital and by the shock of the accident itself, is a point on which a pronouncement is not very easy. In any case it is hardly practicable or desirable to order stimulants for every patient entering hospital with an accident, on the chance of warding off delirium tremens. As a complication of a fracture this condition is a very unfavourable one indeed. The constant restlessness, the attempts to get out of bed, and the utter disregard for the condition of the injured part, from which indeed he will frequently remove surreptitiously the splints, tell very greatly against the chances of a patient who exhibits this combination. In every such case a careful note of the symptoms should be entered in the registers for future production if necessary, and a male special nurse is, of course, always ordered as a precautionary measure against attempts at suicide, murder, or other forms of delirium-excited violence.

As regards treatment, the difficult question to decide is always that of stimulation. The patient's heart often flags in a manner which would secure him the immediate and beneficial administration of a dose of brandy in other circumstances; but reliance is better placed upon strychnine in these circumstances when dealing with a case of delirium tremens. Nevertheless, alcohol stimulation need not be regarded as absolutely inadmissible. For the delirium itself bromides in big doses give the best results: hyoscine is not regarded with great favour by many of those who have had experience of its effects. To procure sleep a dose of morphia may be exhibited if the house surgeon is within call for the next few hours, and if he has a reliable nurse in charge of the ward. Otherwise paraldehyde gives as good results as any other hypnotic.

Although the efforts of the medical officer must be devoted to conquering the sleeplessness of the patient, it is not well to allow the administration of any drug for this purpose except after personal inspection of the patient. To leave instructions, for example, with the night nurse to give such and such a dose at midnight, and so much more at three o'clock if the man has not slept, is not good treatment: for in many of these cases absorption from the intestinal tract takes place very slowly, and the cumulative effect of several successive doses of powerful drugs is a possibility which must never be left out of mind.

A patient with delirium tremens must be liberally fed, notwithstanding the fact that his digestion is likely to be poor, owing to gastritis, hepatic insufficiency, or other causes. For the reason, doubtless, that appetite is stimulated thereby, a liberal allowance of stout with meals has often been recommended as a successful line of treatment, and certainly many cases seem to do well when thus prescribed for. Even more to the point is a sufficient preliminary dose of calomel, followed by a regular saline aperient each morning: this is all the more necessary if opium in any form has been ordered to combat restlessness or insomnia.

